# Supplementary material for: Patterns of Intron Gain and Loss in Fungi
Source: PLoS Biol. 2004 Nov 30;2(12):e422. doi: 10.1371/journal.pbio.0020422 (PMC532390; doi:10.1371/journal.pbio.0020422)
Supplement: Table S1 — Also available at http://genes.mit.edu/NielsenEtAl/. (4.3 MB ZIP). [file pbio.0020422.st001.zip › NielsenEtAl/html/848.html]

AN5574.1.NCU09561.1.MG00213.1.FG04453.1


```
 CLUSTAL W (1.82) Multiple Sequence Alignments - Introns Inserted


Sequence 1: MG00213.1	207 aa
Sequence 2: FG04453.1	179 aa
Sequence 3: NCU09561.1	218 aa
Sequence 4: AN5574.1	152 aa
Alignment Length: 218 aa
Number Identitical Residues: 48 aa
Alignment Score (without introns) 3242


MG00213.1 	MSYYDIDAILTDAE0KVPCEFQLDVPELGYLDNTPSHA~LKAGTKVNLPLWLAEMLALAN
NCU09561.1	MSYYDIDAILTDAE0KIPCTFQIDVPDLGYLDNQPGHT~LKSGSRVALPIWLAEMLAIAN
FG04453.1 	MSYYDIDAILTDAE0KVPCQFEIDVPYLGHLDNSP-NG~LKANTPLTLPLWLAEMLALAS
AN5574.1  	MSYYDIDSILTDAQ0KLPCTFELDVPGLGILEGNPGES0ADPRT--------VDLRSLAP
          	*******:*****: *:** *::*** ** *:. *..   .. :        .:: ::* 

MG00213.1 	TTHPSSADPDDA-RSFVSLNLPNPLADDVIQALKADPRSVPLRDRSPHFFSLSTRVLDLF
NCU09561.1	TGAVDMDDPSQSSKSFITFDLPPALGNDVVQALKADPRSVPLRDQSAHFYALATHMMELS
FG04453.1 	T--PTSNSP-------LTLNLPPCLSTAVLSALKADPRAVPLRDHSLHFYGVGVRMLDLF
AN5574.1  	N--------------------YYRLSERVLELFEEEEMVDILSN-------VTTPTLQTS
          	.                       *.  *:. :: :     * :       : .  ::  

MG00213.1 	EERELASVLRKSFVVRAGDVGLHARKVGGGG----------GGHNAGGRGKDDDKGSNIG
NCU09561.1	EEPELSAVLRKTFVSRAAEIALHARKVGGGGSSYHGRDGGGAGGKGKGKATKDDNASNLG
FG04453.1 	DEKDVAEVLRKTFVVRAGDVGLHARKADEG-----------------------------M
AN5574.1  	LDAALLTRTDQTFKKRAAEIADHAHNPKGAL-----------------------------
          	 :  :     ::*  **.::. **::   .                              

MG00213.1 	LGGQSEEFLRGLDEWERNL~FRKAHEGTKAGGEWMDSVKKN
NCU09561.1	VGGAGEDFLRGLDEWERKL~FRSAHDGTKASKEWMENVKKR
FG04453.1 	VGGNGEEFLRGLEEWERGL~FRRGHEGVKGAKEWTDKVKKT
AN5574.1  	--GDGVEFLQGLDETERQL1FRVAHDGARETRIWAGEAKKR
          	  * . :**:**:* ** * ** .*:*.:    *  ..**
```
